# Quantifying Variability of Avian Colours: Are Signalling Traits More Variable?

**DOI:** 10.1371/journal.pone.0001689

**Published:** 2008-02-27

**Authors:** Kaspar Delhey, Anne Peters

**Affiliations:** Max Planck Institute for Ornithology, Vogelwarte Radolfzell, Radolfzell, Germany; University of Exeter, United Kingdom

## Abstract

**Background:**

Increased variability in sexually selected ornaments, a key assumption of evolutionary theory, is thought to be maintained through condition-dependence. Condition-dependent handicap models of sexual selection predict that (a) sexually selected traits show amplified variability compared to equivalent non-sexually selected traits, and since males are usually the sexually selected sex, that (b) males are more variable than females, and (c) sexually dimorphic traits more variable than monomorphic ones. So far these predictions have only been tested for metric traits. Surprisingly, they have not been examined for bright coloration, one of the most prominent sexual traits. This omission stems from computational difficulties: different types of colours are quantified on different scales precluding the use of coefficients of variation.

**Methodology/Principal Findings:**

Based on physiological models of avian colour vision we develop an index to quantify the degree of discriminable colour variation as it can be perceived by conspecifics. A comparison of variability in ornamental and non-ornamental colours in six bird species confirmed (a) that those coloured patches that are sexually selected or act as indicators of quality show increased chromatic variability. However, we found no support for (b) that males generally show higher levels of variability than females, or (c) that sexual dichromatism *per se* is associated with increased variability.

**Conclusions/Significance:**

We show that it is currently possible to realistically estimate variability of animal colours as perceived by them, something difficult to achieve with other traits. Increased variability of known sexually-selected/quality-indicating colours in the studied species, provides support to the predictions borne from sexual selection theory but the lack of increased overall variability in males or dimorphic colours in general indicates that sexual differences might not always be shaped by similar selective forces.

## Introduction

It is usually acknowledged that variation in sexually selected traits is greater than that in comparable naturally selected traits [1]. In fact, the presence of high variance in an extravagant phenotypic trait is often interpreted as evidence for it being sexually selected. On the other hand, sexual ornaments are usually subject to strong, directional selection, which should result in depletion of available (genetic) variability [2], [3]. This apparent discrepancy between theoretical expectation and empirical data, termed the paradox of the lek, has pre-occupied evolutionary biologists for decades [2]–[7]. Recently, a solution has been proposed based on the contention that sexually selected traits show higher condition-dependent expression than non-ornamental traits [6]. The evolution of condition-dependent expression of ornamentation may maintain phenotypic and genetic variability in sexually selected traits as condition itself is expected to have high genetic variance. This variability is unlikely to be depleted by directional selection as variability in condition is probably determined by variation in multiple loci dispersed over the whole genome [6].

Empirical studies that revealed considerable variation in ornamental (sexually selected) traits, often exceeding that found in putatively naturally selected traits, have been instrumental in the development of new theoretical models. However, their conclusions are based on a limited set of traits since comparisons have by and large focused on metric traits such as the size of elongated tail feathers in birds [5], [8]–[10], or eye stalk length in flies [11]. Similar studies on other types of sexual traits are largely missing, an important deficit since patterns of variability may differ between different trait types [8]. Particularly ill studied in this regard is variability in coloration [5], [12], although colours constitute currently some of the best examples of sexually selected traits, especially in birds [13].

Apart from the greater effort and more extensive equipment required to derive objective measurements of coloration (reflectance spectra) compared to metric traits, this omission is most likely largely due to computational difficulties to compare variability in coloration. Unlike metric traits, coefficients of variation are unsuited to estimate variability in coloration because colours are often quantified using arbitrary scales and thus their variance does not scale with the mean [12]. Hence, direct comparisons with putatively naturally selected metric traits (such as tarsus length) are flawed. An alternative would be to compare variability between sexually and naturally selected colours [12]. This has rarely been attempted [but see 14], because different colours are usually described on different scales that are also not directly comparable [see review in 15].

Here we quantify the degree of variation in bright and drab colour patches in six common and well-studied European passerine birds by implementing current models of avian colour vision [16]. Specifically we aim to test the main prediction of the condition-dependent handicap models of sexual selection [11], [17] namely that (a) sexually selected/quality-indicator traits should show amplified variability compared to equivalent non-sexually selected traits. In addition we tested the ensuing prediction that (b) males should be more variable than females, given that they are usually the sexually selected sex. Finally, since the degree of sexual dichromatism is often used as a proxy for sexual selection we also tested the prediction that (c) sexually dimorphic traits should be more variable than monomorphic traits.

## Materials and Methods

### Study species

Individuals of six passerine birds (blackcap [*Sylvia atricapilla*], European robin [*Erithacus rubecula*], blue tit [*Cyanistes caeruleus*], great tit [*Parus major*], blackbird [*Turdus merula*] and greenfinch [*Carduelis chloris*]) were captured in mist nets in the surroundings of Möggingen (47°75′N, 9°07′E), Germany between March and June 2005 (see Table 1 for sample sizes). Our aim was to estimate the amount of variability in coloration, as perceived by the birds, that would be available for mate choice or rival assessment in a given season and population. We chose to work with live birds instead of museum specimens to avoid introducing other sources of variation that may obscure patterns of variability. In addition to the potential that plumage colours may fade with specimen age, of particular concern are biological sources of variation such as non-systematic differences between years, and differences between collection sites. Nevertheless, museum specimens may constitute valuable sources of data to estimate colour variability especially if it can be shown that patterns of variability broadly agree with those found using wild birds, as in the present study. The species sampled were selected because they provide a diverse array of colours (structural, melanin- and carotenoid-based) and because they are common in the study area, allowing us to obtain the sample sizes that are required to estimate trait variability. The time frame of capture was chosen to include the reproductive season when sexual signalling is presumably intense. All target species are mainly socially monogamous, although low levels of polygyny have been recorded. When unambiguous, birds were sexed by external traits (blackcaps, great tits, blackbirds). Robins, blue tits and greenfinches were sexed using molecular markers [18]–[21]. To identify known sexually and non-sexually selected or quality indicator colours we performed a review of the literature on putative signalling functions of any colour in all study species (Text S1). From this review it became clear that, although these species have been intensively studied, it is not always possible to obtain unambiguous evidence suggesting that a particular colour patch is sexually selected (favoured through agonistic interactions between rivals or through mate choice). Thus, we decided to include also plumage patches where colour expression acts as an indicator of quality or shows condition-dependence. These kinds of traits are usually assumed or hypothesized to convey honest information about the quality of their bearers to potential rivals or mates [22], and thus are likely to be sexually selected and show high variability as well.

**Table 1:** List of species used in the this study indicating sample size, measured colour patches and their human-perceived colours, probable colour production mechanism, probable signaling function as described in the literature and level of chromatic (ΔS_sex_) and achromatic (ΔL_sex_) sexual dimorphism.

Species	Sample Size		Patch	Human perceived colour	Colour prod. mechanism	Probable signaling function	Sex. dimorph. (jnd)	
	males	females					ΔS_sex_	ΔL_sex_
**Robin**	16	15	Back	Brown-grey	Melan.	?	0.52	0.68
***Erithacus rubecula***			Breast	Rusty-red	Melan.	Ag.-inter.(?)	1.67	0.17
***Blackbird***	30	10	Head	Black (males), brown (females)	Melan.	?	9.21	13.57
***Turdus merula***			Back	Black (males), brown (females)	Melan.	?	5.56	8.99
			Breast	Black (males), brown (females)	Melan.	?	8.71	17.4
			Bill	Yellow-orange	Carot.	Q-indic., Ag.-inter., M-choice(?)	9.44	6.8
**Blackcap**	44	22	Head	Black (males), rusty-red (females)	Melan.	?	16.9	22.08
***Sylvia atricapilla***			Back	Brown-grey	Melan.	?	1.48	0.59
			Breast	Grey	Melan	?	2.74	0.88
**Great tit**	27	23	Head	Black	Struct.+Melan.	Q-indic., M-choice	5.81	3.37
***Parus major***			Back	Green	Carot.+Melan.	?	1.59	1.39
			Breast	Yellow	Carot.	Q-indic.	0.72	3.47
			Cheek	White	Struct	?	0.91	1.08
**Blue tit**	20	17	Head	Blue	Struct.	Q-indic., Ag.-inter., M-choice	5.25	2.0
***Cyanistes caeruleus***			Back	Grey-green	Carot.+Melan.	?	2.68	1.53
			Breast	Yellow	Carot.	Q-indic.	1.72	1.53
			Cheek	White	Struct.	?	1.71	1.08
**Greenfinch**	41	20	Head	Grey-green	Carot.+Melan.	?	4.49	2.68
***Carduelis chloris***			Back	Grey-green	Carot.+Melan.	?	3.47	2.43
			Rump	Green-yellow (males), green (females)	Carot.+Melan.	?	2.26	1.54
			Tail	Yellow	Carot.	Q-indic.	5.27	3.46
			Breast	Green-yellow (males), brown-green (females)	Carot.+Melan	Q-indic.	8.10	1.87

Colour production mechanisms (melanin-, carotenoid-based, structural colours and combinations thereof) were collated from the literature when known (see Text S1) or determined based on the shape of reflectance spectra following Doucet et al. [61]. Probable signaling function was categorized as: Q-indic. ( = quality indicator, the expression of colour correlates with aspects of individual quality such as condition, health, parental abilities, etc), Ag.-inter ( = agonistic interaction, expression of the colour determines or influences the outcome of aggressive interactions), and M-choice ( = mate choice, male colour expression determines female preferences, measured by traits such as date of egg-laying, paternity, brood sex ratios, differential allocation patterns, etc.). Bibliographic references in support of the probable signaling function of each coloured patch are given in Text S1.

### Reflectance spectrometry

Plumage reflectance of different plumage patches (see Table 1) was measured using an Avaspec 2048 spectrometer connected to a deuterium-halogen light source (Avalight-DHS, Avantes, Eerbek, Netherlands) through a bifurcated fibre optics cable fitted at the end with a plastic cylinder to standardise measuring distance and shield out ambient light. The probe was held perpendicular to the surface of the feathers (or bill in the case of the blackbird) hence illumination and recording angles were both 90°. Reflectance was computed relative to a WS-2 white standard using the program Avasoft 6.2.1. We took a set of five reflectance readings of different predefined and standardized spots in each body part (Table 1). Reflectance values between 300 to 700 nm (in 1 nm steps) were imported into custom made spreadsheets for further analysis. Average reflectance spectra for each species, patch and sex are given in Fig. S1.

### Visual modelling

Most diurnal birds present six types of photoreceptors in their retinas, four types of single cones, double cones and rods [23]. While rods are used for vision in low light levels, and double cones (composed of two cells in close electrical and physical contact) are thought to mediate achromatic tasks (luminance or brightness perception), colour vision in diurnal birds depends on the four types of single cones, that are sensitive to very short (VS), short (S), medium (M), and long (L) wavelengths respectively [24]. For each reflectance spectrum we computed cone quantum catches (Q_i_) for each cone type using the formula:
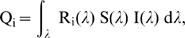
(1)where λ indicates wavelength, R_i_(λ) the sensitivity of the cone type, S(λ) the reflectance spectrum, and I(λ) the spectrum of irradiant light [16]. Vision of passerine birds is chiefly differentiated by the sensitivity maxima of the VS cone, with other cone sensitivities being similar. The species included in the present study all belong to the Passerida, which according to comparative molecular analysis of the opsin gene sequence have U-type eyes with peak sensitivity of the VS cone at 367 nm [25], [26], which has been confirmed through microspectrometry for blue tit and blackbird [27]). Therefore we used generalized spectral cones sensitivities of U-type birds [from Appendix 1 in 26].

**Figure 1:**
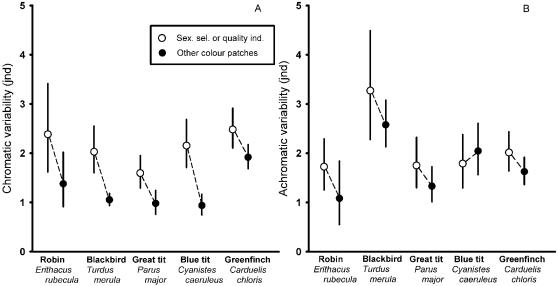
Discriminable chromatic (ΔS_var_, A) and achromatic variability (ΔL_var_, B) of sexually selected or quality indicator colour patches versus other colour patches in males of five species of European birds. Depicted are means and 95% confidence intervals (back transformed after Box-Cox transformation prior to analysis).

Relative (each cone quantum catch divided by the sum of all four) cone quantum catches can be plotted (after mathematical transformation according to [28]) in a three-dimensional tetrahedron where each vertex represents the sole stimulation of a different cone type. Thus, measurements of differently coloured patches are represented by clouds of points in the avian visual space (see Fig. S2). In general the smaller the Euclidean distance between two points in this space, the smaller the difference in visual contrast between the corresponding reflectance spectra, and below a certain threshold distance two spectra will no longer be discriminable. These thresholds are determined by receptor noise, which varies with cone type [16], [29]. Using this model we calculated chromatic discriminability (ΔS) between two points in the tetrahedral space following the equation:
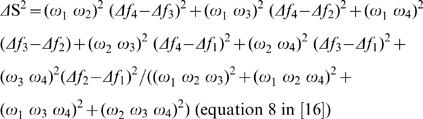
(2)where
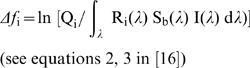
(3)and S_b_(λ) represents the reflectance spectrum of the background (brown bark, see Fig. 1B online appendix), ω_i _represents receptor noise [16] that was computed using a Weber fraction of 0.05 and cone proportions of 1∶1∶2∶2 (VS:S:M:L; [26]). The Vorobyev-Osorio model we used assumes that colour discriminability depends only on receptor noise and that differences in intensity (i.e. brightness or luminance) are disregarded [16]. This model accurately predicts colour discrimination ability in birds, bees and humans [29] and has been used for example to estimate sexual dichromatism [30] and detectability of birds and fruit to other avian predators and frugivorous birds respectively [31], [32].

We also quantified variation in brightness or luminance. Achromatic variation in birds is probably detected by the double cones [33], [34]. We used equations (1) and (3) to compute double cone quantum catches using double cone spectral sensitivity data from *Leiothrix lutea* provided by Martin Schaefer [35]. The achromatic contrast between two spectra can be computed as:
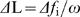
(4)where ω = 0.05 [36]. For more details on visual modelling see Vorobyev et al. [16] and Siddiqui et al.[36].

The unit for ΔS and ΔL is the jnd (just noticeable difference) and values of >1 can be discriminated by birds, whereas those below this threshold cannot [16]. To estimate the degree of discriminable variation in coloration within each plumage patch for our sample we computed the visual contrast (hereafter ΔS_var_ or ΔL_var_) between each point and a fixed point in space. The chosen point was the joint mean of each cone quantum catch for ΔS_var_ and the mean double cone quantum catch for ΔL_var,_ computed separately for each species, sex and plumage patch. Note that this procedure is analogous to a Levene's test for the unequality of variances. Samples with high discriminable variability in coloration should have large mean values of ΔS_var_ and/or ΔL_var_. Thus, this measurement should provide us with a proxy of how much discriminable variability in coloration is available for assessment to potential mates or rivals. The degree of sexual dichromatism for each colour patch was estimated as ΔS or ΔL between the average point of each patch of males and females (as in [30], hereafter ΔS_sex_ or ΔL_sex_). For a graphical representation of the visual modelling procedure and computation of ΔS_var_ and ΔS_sex_ see Fig. S3.

The results of the Vorobyev-Osorio model may be influenced by variation in biologically relevant parameters, such as background and type of irradiant light. Neither ΔS nor ΔL values change with background type if we assume that all birds are seen against the same background [data not shown, see also 30]. Variation in irradiant light, on the other hand, may affect ΔS or ΔL, even when all birds are illuminated by the same light [see for example 16]. Thus we repeated all analyses using the following irradiances: forest and woodland shade (measured in the study site, see Fig. S4) and uniform irradiance [as in 30]. Forest shade is typical for the under storey of forests were the light is filtered by green leaves and is rich in intermediate and long wavelengths while woodland shade is found in forest gaps were the direct light from the sun is blocked by the trees, being rich in short wavelengths [37]. These irradiance types thus represent realistic (forest and woodland shade) light environments while their different spectral properties allows us to validate the robustness of the results. Using different irradiances had only small effects on the analyses and the main conclusions of the study are unaffected by the type of illuminant used in the models. Below we present the data using D65 as the sole illuminant but we provide the results for all analyses in Tables S1, S2 and S3.

### Statistical analysis

The distribution of ΔS_var_ and ΔL_var_ generally did not follow a normal distribution and therefore we Box-Cox [38] transformed the data prior to analysis. To assess differences in ΔS_var_ and ΔL_var_ between patches and sexes we used ANOVA including the factors sex, patch and their interaction in the model. If the interaction term was significant we analysed both sexes separately, if not, the interaction term was removed before testing for main effects [39].

Despite the large number of studies on coloration in our target species, published evidence for evolutionary significance of colours is only available for colour patches shown to be sexually selected or indicators of quality (Table 1). In agreement with the general paucity of studies addressing evolutionary significance of drab or cryptic coloration, there appear to be no published studies addressing the signalling function of putatively naturally selected traits in our study species. Therefore, for the purpose of our comparison between sexually and naturally selected colours, we compare ΔS_var_ and ΔL_var_ between those patches that have been demonstrated to be important in sexual selection or that are known indicators of individual quality, and those for which no such information is available. This analysis includes only males since there is even less information available for females. See Text S1 for a summary of the evidence for the different colour patches.

To test the hypothesis that the degree of sexual dichromatism in a patch is associated with colour variability we used ordered heterogeneity tests [O-H, 40]. This test is based on the (common) assumption that sexual dichromatism is a valid proxy for the intensity of sexual selection [e.g.41], [42]. The composite statistic r_s_P_c_ was computed following Rice and Gaines [40], where P_c_ is the complement of the p value (1-p) obtained for the factor “patch” in the ANOVAs (Table 2). To obtain r_s_P_c_, P_c_ is multiplied by the Spearman's rank correlation coefficient (r_s_) obtained by correlating ΔS_var_ or ΔL_var_ with sexual dichromatism (ΔS_sex _or ΔL_sex_ respectively) across coloured patches. One-tailed p-values for r_s_P_c_ were obtained from Fig. 1 in [40]) where k represents the number of different coloured patches measured. O-H tests were performed separately for each species, if the interaction term sex*patch reached significance we computed the tests separatedly for males and females, otherwise the sexes were pooled. O-H tests were not performed for robins as they are redundant given that only two coloured patches were measured.

**Table 2:** Results of the ANOVAs testing for sex and patch differences in discriminable chromatic (ΔS_var_) and achromatic (ΔL_var_) variability and corresponding Ordered Heterogeneity tests testing for a positive relationship between levels of variability and sexual dichromatism.

		sex	patch	sex x patch	Ordered heterogeneity tests sexual dichromatism vs. variability
**Robin** *Erithacus rubecula*	ΔS_var_	F_1,59_ = 0.02, p = 0.88	**F_1,59_ = 11.07, p = 0.0015**	F_1,58_ = 0.54, p = 0.46	1)
	ΔL_var_	F_1,59_ = 0.39, p = 0.546	F_1,59_ = 1.53, p = 0.22	F_1,58_ = 0.8, p = 0.389	1)
**Blackbird** *Turdus merula*	ΔS_var_	F_1,155_ = 2.93, p = 0.088	**F_3,155_ = 10.08, p<0.0001**	F_3,152_ = 2.01, p = 0.114	**r_s_P_c_ = 0.99, k = 4, p<0.001**
	ΔL_var_	**F_1,155_ = 4. 54, p = 0.0347**	F_3,155_ = 2, p = 0.115	F_3,152_ = 0.8, p = 0.524	r_s_P_c_ = −0.70, k = 4, p>0.95
**Blackcap** *Sylvia atricapilla*	ΔS_var_	**F_1,194_ = 8.78, p = 0.0034**	**F_2,194_ = 3.57, p = 0.026**	F_2,192_ = 2.81, p = 0.062	r_s_P_c_ = 0.48, k = 3, p>0.05
	ΔL_var_	F_1,194_ = 2.17, p = 0.142	F_2,194_ = 2.69, p = 0.070	F_2,192_ = 2.03, p = 0.331	r_s_P_c_ = 0.46, k = 3, p>0.1
**Great tit** *Parus major*	ΔS_var_	F_1,195_ = 0.44, p = 0.5	**F_3,195_ = 24.38, p<0.0001**	F_3,192_ = 2.56, p = 0.056	r_s_P_c_ = −0.39, k = 4, p>0.8
	ΔL_var_	F_1,195_ = 1.16, p = 0.281	F_3,195_ = 1.22, p = 0.301	F_3,192_ = 1.14, p = 0.331	r_s_P_c_ = 0, k = 4, p = 0.5
**Blue tit** *Cyanistes caeruleus*	ΔS_var_	F_1,143_ = 0.38, p = 0.53	**F_3,143_ = 21.38, p<0.0001**	F_3,140_ = 1.57, p = 0.19	r_s_P_c_ = 0.39, k = 4, p>0.1
	ΔL_var_	F_1,143_ = 0.22, p = 0.639	**F_3,143_ = 5.99, p = 0.0007**	F_3,140_ = 1.68, p = 0.172	r_s_P_c_ = 0.19, k = 4, p>0.2
**Greenfinch** *Carduelis chloris*	ΔS_var_	---------	**Males: F_4,200_ = 3.07, p = 0.0173**	**F_4,295_ = 4.75, p = 0.001**	**Males: r_s_P_c_ = 0.59, k = 5, p<0.05**
			**Females: F_4,95_ = 10.10, p<0.0001**		Females: r_s_P_c_ = 0.50, k = 5, p>0.05
	ΔL_var_	---------	**Males: F_4,200_ = 5.07, p = 0.0007**	**F_4,295_ = 2.85, p = 0.024**	Males: r_s_P_c_ = 0.099, k = 5, p>0.4
			**Females: F_4,95_ = 4.87, p = 0.0013**		Females: r_s_P_c_ = 0.49, k = 5, p>0.05

Significant terms are depicted in bold.

1) Ordered heterogeneity tests were not computed for robins as only two patches were measured. In this case chromatic variability (ΔS_var_) was higher for the more sexually dichromatic patch (breast) as indicated by Figure 1A, Table 1, and the significant “patch” factor; this was not the case for achromatic variability (ΔL_var_) where there was no significant difference in variability between the two patches (Fig. 1G).

Residuals of the final models did not significantly depart from normality except for the ANOVA on ΔL_var_ for the greenfinch (depicted in Table 2) and the comparison of ΔS_var_ between sexually selected and putatively non-sexually selected traits in the blackbird. In both cases the Shapiro-Wilk test indicated slight departures from normality (p = 0.048 and 0.044 respectively). Statistical tests were carried out with JMP 5.1.

## Results

### Chromatic variability: sexually-selected/quality-indicator patches

**Figure 2:**
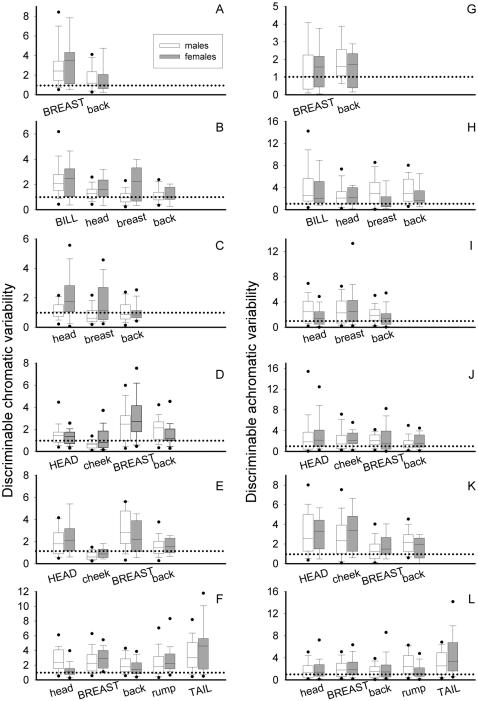
Discriminable chromatic (ΔS_var_, left) and achromatic variability (ΔL_var_, right) of coloured patches for six species of European birds. Robin (A, G), blackbird (B, H), blackcap (C, I), great tit, (D, J), blue tit (E, K) and greenfinch (F, L). Depicted are medians, 25^th^ and 75^th^ percentiles (boxes), 10^th^ and 90^th^ percentiles (whiskers) and 5^th^ and 95^th^ percentiles (dots). Coloured patches that have been shown to be sexually selected or indicators of quality are written out in upper case font (see Table 1 for more information). The dotted horizontal line indicates the 1 jnd discriminability threshold.

Variability in coloration was higher for those colour patches for which there is evidence of being sexually selected or indicators of quality when compared with the rest (see Table 1 and Figs. 1 and 2, computed for males only: robin, F_1,30_ = 4.59, p = 0.0403; blackbird, F_1,118_ = 31.17, p<0.001; blackcap, no data available; great tit, F_1,106_ = 9.40, p = 0.0028; blue tit, F_1,78_ = 27.54, p<0.001; greenfinch, F_1,203_ = 6.18, p = 0.0137). Patches shown to be sexually selected or quality indicators showed on average 0.87 jnd (range = 0.56 to 1.21 jnd) higher discriminable variability when compared to the rest of the coloured patches in the five studied species (Fig. 1). Results for all illuminants yielded similar results and are presented in Table S1.

### Chromatic variability: sex differences

In all species there were significant differences in variability between patches (Fig. 2, Table 2) that followed broadly the same pattern in both sexes, with equivalent colour patches being the most variable in males and females (except in the greenfinch, see below). In general there was no evidence that male colours were more variable: colours in males showed similar levels of variability as in females. There was one exception: female colours in the blackcap were more variable than male colours, and there was a similar trend in blackbirds (Table 2). For the greenfinch the sex*patch interaction reached significance. Analysing males and females separately in this species revealed that tail colour showed the highest variability in both sexes but that the order of variability in the other patches was different (Fig. 2). Results for all illuminants yielded similar results and are presented in Table S2.

As the analysis above included all patches, also those with low sexual dichromatism, we repeated the analysis comparing ΔS_var_ between sexes for the most sexually dichromatic patch in each species (head in blue tits, great tits and blackcaps, bill in blackbirds, tail in greenfinches and breast in robins). Results were unchanged and sex differences were only significant for blackcaps, female (rufous) head colour being more variable than male (black) head colour (Fig. 2; robin_[breast]_: F_1,29_ = 0.16, p = 0.689; blackbird_[bill]_: F_1,38_ = 0.03, p = 0.862; blackcap_[head]_: F_1,64_ = 4.70, p = 0.0339; great tit_[head]_: F_1,48_ = 0.28, p = 0.598; blue tit_[head]_: F_1,35_ = 0.60, p = 0.441; greenfinch_[tail]_: F_1,59_ = 1.29, p = 0.259) . We repeated these tests for each species, sex, patch and illuminant used and the conclusions remained unchanged. These results along with means and 95% confidence intervals are provided in Table S3.

### Chromatic variability: sexually dimorphic vs. monomorphic patches

Within-species, coloured patches with higher sexual dichromatism were more variable in the robin, blackbird and male greenfinches but not in blackcaps, great tits, blue tits and female greenfinches, as indicated by the ordered heterogeneity tests (Table 2). Results for all illuminants yielded similar results and are presented in Table S2.

### Achromatic variability: sexually-selected/quality-indicator patches

Achromatic variability tended to be higher (average = 0.38 jnd, range = −0.25 to 0.69 jnd) for those coloured patches shown to be sexually selected or indicators of quality compared to the rest in all species except for the blue tit (Table 1 and Figs. 1 and 2) but these differences were not significant (computed for males only; robin: F_1,30_ = 2.49, p = 0.124; blackbird: F_1,118_ = 1.7, p = 0.194; blackcap: no data; great tit: F_1,106_ = 1.94, p = 0.16; blue tit: F_1,78_ = 0.47, p = 0.492; greenfinch: F_1,203_ = 2.64, p = 0.105). Results for all illuminants yielded similar results and are presented in Table S1.

### Achromatic variability: sex differences

Differences in achromatic variability between patches were much less marked than in ΔS_var_ (Fig. 2) and only significant for the blue tit and greenfinch (Table 2). In general there were no significant differences in achromatic variability between sexes with the exception of the blackbird, where males seemed more variable than females. The sex*patch interaction was again only significant for the greenfinch. Results for all illuminants yielded similar results and are presented in Table S2.

Comparing variability of males and females for the most sexually dimorphic patch yielded in general similar results as in most cases these differences were not statistically significant, with the exception of the blackcap and blackbird where males were more variable than females (robin_[back]_: F_1,29_ = 0.02, p = 0.871; blackbird_[bill]_: F_1,38_ = 4, p = 0.052; blackcap_[head]_: F_1,64_ = 4.44, p = 0.038; great tit_[breast]_: F_1,48_ = 1.01, p = 0.319; blue tit_[head]_: F_1,35_ = 0.02, p = 0.884; greenfinch_[tail]_: F_1,59_ = 2.85, p = 0.096). We repeated these tests for each species, sex, patch and illuminant used and the conclusions remained unchanged. These results along with means and 95% confidence intervals are provided in Table S3.

### Achromatic variability: sexually dimorphic vs. monomorphic patches

Sexually dimorphic patches were not more variable as indicated by the non significant ordered heterogeneity tests (Table 2). Results for all illuminants yielded similar results and are presented in Tables S2.

## Discussion

The main findings of the present study can be summarized as follows: we showed that (a) those coloured patches for which there was published information suggesting a sexual signalling or quality-indicator function showed higher levels of variability than the rest (chromatic variability only). Nonetheless, (b) males did not consistently show higher colour variability across species (chromatic and achromatic variability). Finally, (c) the data provided only limited support for the prediction that more sexually dimorphic colour patches are generally more variable than monomorphic colours (chromatic and achromatic variability).

### Are sexually-selected/quality-indicator colours more variable?

The ideal test of the predictions of elevated variability in sexually selected colours would be a comparative analysis of variability of all sexually or naturally selected colour patches in a number of species. We developed an index that quantifies variability of different colours on comparable scales, thereby overcoming previous computational difficulties to perform such an analysis. However, information on signaling functions of, or selection pressures on, colour appears only available for patches that look conspicuous to the human eye, which are thus assumed to be important in signalling (Table 1). Similar information on more subtle colours, such as the brown and green colours that are common in many species, is lacking. Therefore we compared variability of those coloured patches known to be sexually selected or indicators of quality with the other measured patches. This comparison demonstrated higher levels of chromatic (but not achromatic) variability in the former, confirming the assumption that sexual selection may be associated with especially variable traits. This result however, should be considered preliminary for two reasons. First, future studies may show that some of the hitherto unstudied coloured patches may also have a function in sexual signaling, and second, we assumed that quality-indicator or condition-dependent colour traits are also sexually selected which may not always be the case. Clearly, more work is needed to be able to confirm that sexually selected colours are more variable than comparable traits, and we hope that the method and results we describe here may stimulate further research in this area.

Interestingly, our results were largely unaffected when using four different types of light environments (see Table S1). This indicates that variability in environmental light conditions, although potentially affecting conspicuousness of birds [e.g. 43], does not greatly affect the degree of discriminable variability between individuals due to colour-constancy, which has been described as the ability of perceiving a given reflectance spectrum as a fixed “colour” under variable illumination [16]. This suggests that a female, for instance, does not gain more or different information by assesing potential mates under different illuminants. The relative insensitivity of chromatic variability to changes in environmental light may be also the reason why chromatic contrast is used for object quality recognition while on the other hand, achromatic contrasts are used for shape recognition and movement detection [33]. This may also explain the lack of consistent differences in achromatic variability between colour patches and between sexually selected and non-sexually selected colours.

### Variability and sexual dichromatism

Sexual dichromatism in birds is often thought to be linked to sexual selection intensity [41], [42]. However, although known sexually selected patches showed increased variability, our analysis did not reveal a consistent relationship between variability and sexual dimorphism in coloration. This suggests (based only on patterns of variability) that sexual dichromatism is not always a very precise proxy for sexual selection. Sexual dimorphism in some coloured patches could have arisen due to natural instead of sexual selection (see [44], [45]) or through a combination of both, for instance when habitat differences drive divergence in appearance between the sexes [46]. Additionally, not all sexually dichromatic patches are necessarily quality signals, they may also function to indicate sex, and such signals are likely to be highly optimised and invariant [12]. Alternatively, some naturally selected colours may show genuine high levels of variability. Colours that probably have a camouflage function, for instance the brown-green back plumage in most of the studied species, may show high variability if the background against which they have to blend is highly heterogeneous [12], [47]. Meanwhile, before we can make further progress, we need more information on selection pressures on, and condition-dependence of, dichromatic and monochromatic colours.

### Sex differences in variability

We had predicted that males should show higher levels of colour variability than females. The rationale behind this prediction is that sexual signals should show higher levels of condition-dependence (and thus variability) in males than in the corresponding traits in females [17]. Although this prediction is supported by some experimental and correlational studies [11], [48], [49] other researchers have found the opposite pattern (i.e. females being more variable than males, [10], [50]) or no sex difference in variability [9], [51], [52]. Our data seems to mainly add to these last findings since no general increased variability in males was found and, even when including only the most sexually dichromatic patch, males were not generally more variable than females. We suggest that the fact that patterns of variability are broadly similar in males and females might indicate that ornaments may be more often than expected used for mutual mate assessment [53] or be important for status signalling in both sexes, as has been suggested for highly variable morphometric ornaments in females [10]. If these patterns are confirmed in larger datasets it would lend support to the mounting evidence on the importance of female ornamentation [54]. Alternatively, while we show here that females display similar levels of discriminable variability in coloration not all this variability may be equally informative of individual quality. A given amount of variation at the high end of ornament exaggeration (usually males) could provide more information and carry more costs (production costs, detectability to predators) than the same level of variability at the low end (usually females) of ornament exaggeration. This possibility could be assesed by determining the linearity of condition-dependent expression of colours with different levels of exaggeration.

### Concluding remarks

While initially riddled with methodological problems [12] studies of colour variability can now be based on physiological models of colour perception. Indeed, currently we can probably quantify better how birds and other animals may perceive variation in coloration than how they perceive size differences (e.g. in tail length), thus providing fresh insights into the longstanding debate on ornament variability. The described method can be used to quantify variability at the intra-individual, intra-specific and inter-specific levels, opening up exciting new research avenues. For example, the highly variable, sexually-selected/quality-indicator colours were often (in 4 out of 5 species) due to the deposition of carotenoids (Table 1). Possibly carotenoid-based colours show intrinsically higher levels of variability, perhaps due to their hypothesized increased condition-dependence [see 55], but also [56]. Likewise, the increased chromatic and the decreased achromatic variability in brown (phaeomelanin- based) plumage of the female blackbird and blackcap compared to the corresponding black (eumelanin-based) plumage in the males could be directly related to the type of pigment used [57]. Whether different mechanisms of colour production have different intrinsic levels of variability, is an intriguing issue that could be pursued further based on a more extensive sampling across bird species and coloured patches. If some traits (for instance carotenoid-based coloration) show systematically higher discriminable variability than others this may explain why they feature more prominently as sexually selected ornaments [58], as only traits with sufficient discriminable variability can effectively be used by rivals or mates for assessment, and this can determine which traits end up being used for signaling [59]. Finally, would patterns of variability differ in species under more intense sexual selection, such as polygynous or lekking species? Intriguingly, some polygynous species have lower levels of variability in tail length than closely-related monogamous species [10], although this result was not confirmed by more comprehensive comparative analyses [50]. Future comparative analyses of colour variability may help to shed light on these issues.

## Supporting Information

Table S1 Differences in chromatic (ΔS_var_) and achromatic (ΔL_var_) variability between sexually selected or quality indicator colour patches and other colour patches for four different illuminants. Means and 95% confidence intervals have been back-transformed after Box-Cox transformation prior to analysis.
(0.03 MB XLS)

Table S2 Results of the ANOVAs testing for sex and patch differences in discriminable chromatic (ΔS_var_) and achromatic (ΔL_var_) variability and Ordered Heterogeneity tests for the four illuminants used.
(0.03 MB XLS)

Table S3 Indicates level of sexual dimorphism in coloration (ΔS_sex_ and ΔL_sex_), means and 95% confidence intervals levels of chromatic (ΔS_var_) and achromatic variability (ΔL_var_) for males and females and associated F-tests for the four used illuminants. Means and 95%CIs have been back-transformed after Box-Cox transformation prior to analysis.
(0.06 MB XLS)

Figure S1 Average reflectance spectra of coloured integumentary patches of six European birds. Open symbols and dashed lines correspond to males and filled symbols and closed lines to females. Vertical error bars represent standard errors.
(6.31 MB TIF)

Figure S2 Graphical representation of coloured integumentary patches of six species of European birds in the avian visual space. In the tetrahedral visual space each vertex represents the theoretical sole stimulation of one cone type (VS: very short, S: short, M: medium, and L: long wavelength sensitive cones). (A) Tetrahedron and all data points plotted to show general scale of the three axes (x, y, z), where higher values of X represent greater stimulation of the L cone and lower stimulation of the M cone, higher Y values represent greater stimulation of the S cone, and higher values of Z greater stimulation of the VS cone. Note that the data points lie in general low along the Z axis due to the use of the D65 illuminant which is relatively poor in UV wavelengths (see Fig. S4). (B) References, open symbols represent males and closed symbols females. (C) Robin. (D) Blackbird. (E) Blackcap. (F) Great tit. (G) Blue tit. (H) Greenfinch.
(5.71 MB TIF)

Figure S3 Graphic representation of the procedures used to compute ΔS_var_ and ΔS_sex_. Reflectance spectra of birds (in this example head reflectance of three male and three female blue tits) (A) and background (B) are multiplied by the illuminant (C) and cone sensitivities (D, U-type eyes, from Appendix A in [24]) to obtain light adapted cone quantum catches (E, F) using eqs. 1, 2 in [16]. Cone quantum catches can be plotted (after suitable transformation into x, y, z coordinates, see eqs. A8, A9, A10, A11 in [26]) in the avian visual space, represented here by a tetrahedron (G). Points that lie further apart in this tridimensional space are in general more easily discriminable by the birds, but this depends on receptor noise which differs for the four cone types. To estimate variability for males and females we first computed the discriminability (ΔS) between each point and the sex-specific centroid (i.e. the joint average of the four cone quantum catches, [57], represented here with a square) using eqs. 3, 4, 8 in [16]. Values of ΔS were averaged for males and females separatedly to obtain ΔS_var_. Higher values of ΔS_var_ should thus indicate higher chromatic variability. In this hypothetical example note that males lie further apart in the avian visual space than females and that their ΔSvar is accordingly higher. The chromatic discriminability between male and female centroids provides an estimate of the level of sexual dichromatism (ΔS_sex_, see [28]).
(5.61 MB TIF)

Figure S4 Irradiance spectra used to compute chromatic and achromatic variability. D65 is the spectrum of standard daylight [16], while green light and woodland shade are irradiance spectra collected in the study area on June and January 2007 respectively. The dotted line represents uniform irradiance as used in some studies [e.g. 28].
(0.51 MB TIF)

Text S1 Review of evidence of the signaling function of plumage coloration in the six studied species.
(0.13 MB DOC)
