# Supplementary figures and images for: Quantifying Variability of Avian Colours: Are Signalling Traits More Variable?
Source: PLoS One. 2008 Feb 27;3(2):e1689. doi: 10.1371/journal.pone.0001689 (PMC2253496; doi:10.1371/journal.pone.0001689)

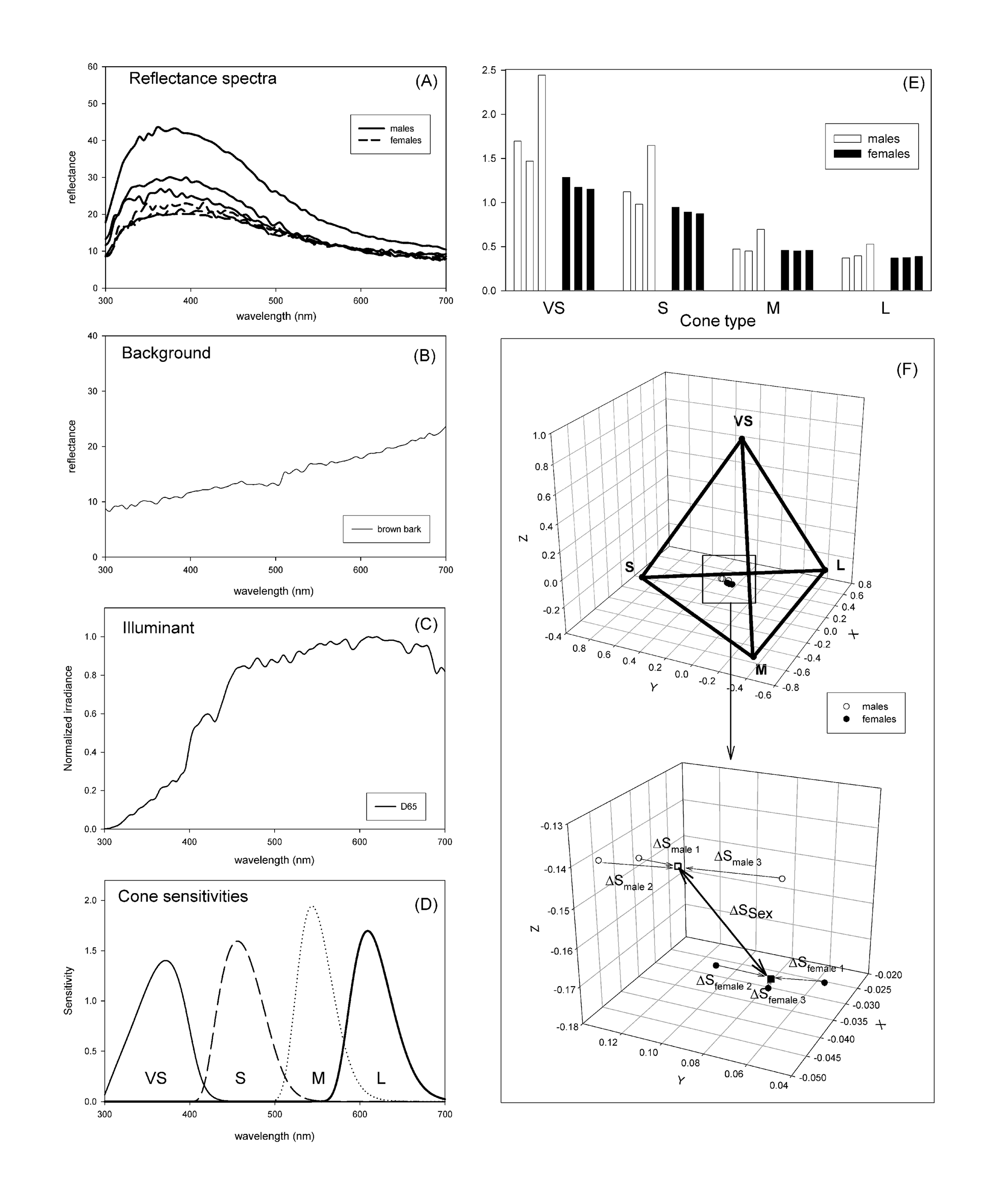

Supplement: Figure S3 — Graphic representation of the procedures used to compute ΔSvar and ΔSsex. Reflectance spectra of birds (in this example head reflectance of three male and three female blue tits) (A) and background (B) are multiplied by the illuminant (C) and cone sensitivities (D, U-type eyes, from Appendix A in [24]) to obtain light adapted cone quantum catches (E, F) using eqs. 1, 2 in [16]. Cone quantum catches can be plotted (after suitable transformation into x, y, z coordinates, see eqs. A8, A9, A10, A11 in [26]) in the avian visual space, represented here by a tetrahedron (G). Points that lie further apart in this tridimensional space are in general more easily discriminable by the birds, but this depends on receptor noise which differs for the four cone types. To estimate variability for males and females we first computed the discriminability (ΔS) between each point and the sex-specific centroid (i.e. the joint average of the four cone quantum catches, [57], represented here with a square) using eqs. 3, 4, 8 in [16]. Values of ΔS were averaged for males and females separatedly to obtain ΔSvar. Higher values of ΔSvar should thus indicate higher chromatic variability. In this hypothetical example note that males lie further apart in the avian visual space than females and that their ΔSvar is accordingly higher. The chromatic discriminability between male and female centroids provides an estimate of the level of sexual dichromatism (ΔSsex, see [28]). (5.61 MB TIF) [file pone.0001689.s006.tif]
