# Supplementary material for: Quantifying Variability of Avian Colours: Are Signalling Traits More Variable?
Source: PLoS One. 2008 Feb 27;3(2):e1689. doi: 10.1371/journal.pone.0001689 (PMC2253496; doi:10.1371/journal.pone.0001689)
Supplement: Text S1 — Review of evidence of the signaling function of plumage coloration in the six studied species. (0.13 MB DOC) [file pone.0001689.s008.doc]

Text S1. Review of evidence of the signaling function of plumage coloration in the six studied species.

**Robin** (*Erithacus rubecula*)

Although the red breast of the robin constitutes a classical example of agonistic signal that elicits aggressive behaviour of territory owners, there are no studies that address the relationship between the degree of colour expression, aggressiveness, outcome of territorial interactions, individual quality or female choice. The closest we could find to such a study would be the work of Chantrey and Workman [1] which states that robin models with red breast elicit a more extreme aggressive behaviour by the territory owner than robin models without a red breast. This could be considered as very weak evidence that the colour of the breast in robins influences agonistic territorial interactions. We are not aware of any study that focuses on the function of the back colour.

**Blackbird** (*Turdus merula*)

While there are several studies on the signaling function of the yellow bill coloration in this species to our knowledge there are no studies on the function of body coloration even though being highly sexually dimorphic.

Bill coloration in the blackbird is produced through carotenoid deposition (α- and β-carotene, β-cryptoxanthin, zeaxanthin and lutein; [2]). Males with more orange coloured bills were paired to females in better condition which produce more young, however, male bill colour itself was not correlated with breeding success [3]. Females did not approach stuffed decoys with orange bills more than decoys with yellow bills [4,5] but females seem to prefer males with intact bill UV reflectance [5]. Males with more orange bills show increased cell-mediated immune responsiveness (PHA-induced swelling) but bill colour was not correlated with the primary response of the humoral immune system (to sheep red blood cells, SRBC) and negatively correlated to the secondary response [6]. However, immunization with SRBC caused bill colour to decline from orange to yellow [2]. Males with more orange bills were larger [7] but did not have less ectoparasites [7]. Evidence supporting a function of bill colour in male-male agonistic interactions is mixed. In a study carried out in France males did not attack or approach differentially male decoys with orange bills compared to decoys with yellow bills [4] while in New Zealand males responded more aggresively to orange-billed models than to yellow-billed models [5].

**Blackcap** (*Sylvia atricapilla*)

We are not aware of any studies focusing on the signaling function of blackcap coloration.

**Great tit** (*Parus major*)

**Head colour:** in a recent study [8] it was shown that UV reflectance of the UV/black crown was positively correlated with condition at moult. Moreover, there was positive assortative mating for this trait.

**Cheek colour:** We have found no study dealing with the expression of cheek coloration in this species. Only one study examined variation in immaculateness (quantified as the degree of raggedness of the border of the patch) of the cheek patch and found correlations with breeding success and agonistic interactions [9]. While the coloration of the cheek patch has not been studied in the great tit, in the related black-capped chickadee (*Poecile atricapillus*) males with brighter cheeks seemed to be preferred by females [10] and had higher reproductive success [11].

**Back colour:** We are not aware of any studies. The greenish back colour is probably due to the combination of carotenoids and melanins [12].

**Breast colour:** The yellow breast coloration of great tits is caused by carotenoid deposition (mainly lutein and zeaxanthin, [13]). Expression of yellow breast coloration is dependent on habitat quality [14]. The intensity of the yellow coloration was positively correlated with the degree of blood parasitism and survival [15]. Breast coloration did not correlate with the levels of antioxidants in blood [16]. Yellower females produced less offspring but were in better condition than duller females while male colour was unrelated to plumage yellowness [17]. Yellow coloration is positively correlated with condition at moult in one study [18] but not in another [8], and not with the levels of flea infestation in nestboxes [19]. However, yellowness of the plumage correlates positively with number of mites in the plumage [20]. Yellower males had higher concentration of heterophil lymphocytes in blood [21]. Males with yellower breast coloration may be better at providing food to offspring [22]. The expression of the breast coloration did not correlate with nest defence intensity [23].

**Blue tit** (*Cyanistes caeruleus*)

**Head colour**: A large body of evidence has been amassed in the last years suggesting that male crown coloration is a sexually selected trait, although different lines of evidence do not always agree. Ultraviolet crown coloration is correlated positively with individual age [24], and coloration correlates positively with survival in a Swedish [25,26] but not in an Austrian population [24]. Correlational and experimental evidence suggest that females overproduce male offspring if paired to highly UV-reflecting males [25-27] but this simple pattern may be more complex and age-dependent in some populations [28] and non-existent in others [29]. Females paired to more UV males also seem to invest differentially more in chick provisioning, nest defence and egg quality [30-32]. Females seem to prefer brighter males and males with intact UV-reflectance in mate choice experiments [33,34] and assortative mating by UV-reflectance in the wild has been reported in one population [35]. Links between crown UV-coloration and paternity success are not as straightforward. While within-pair paternity success seem to be higher for more UV males, at least in some years, extra-pair paternity success was higher for less-UV adult males [36,37]. Experimental colour manipulation however, did not support this patterns as UV-reduced males had lower extra-pair success than UV-increased males [37]. Male UV coloration also correlates with plasma testosterone levels in an age-dependent manner, this correlation being positive for juvenile males and negative for adults [38]. More heterozygous males are more ultraviolet [39], while mite infestation during moult reduces crown coloration [40]. Finally, UV-coloration may play a role in determining the outcome of male-male interactions in some populations but not in others [41-43].

**Cheek colour**: No studies known to us, for data on related species see Great tit.

**Breast colour:** Yellower males seem to be better at raising offspring (better parents) [31,44,45], and have larger fat reserves [46].

**Back colour:** No data available to our knowledge. The greenish back colour is probably due to the combination of carotenoids and melanins [12].

**Greenfinch** (*Carduelis chloris*)

Several studies have addressed the relationship between greenfinch coloration and different measures of success and quality indicators. Unfortunately many of these studies have computed a composite “colourfulness” or “yellowness” score by combining the coloration scores from different body parts [40,47-49] making it impossible to determine whether some coloured patches correlate better with quality or success than others. Below we summarize the data for those few studies that studied coloured patches separatedly.

**Breast colour:** Coloration is based on the deposition of carotenoids (canary-xanthophylls A and B, [50]). The coloration of the breast feathers is affected by coccidiosis during moult [51]. Yellow brightness of the breast feathers correlates positively with the strength of the humoral immune reponse, negatively with heterophil counts and did not correlate with lymphocyte counts, heterophil/lymphocyte ratio, white blood cell counts or cellular immune response [52]. In another study breast chroma was correlated negatively with humoral immunocompetence [53].

**Tail colour:** Coloration is based on the deposition of carotenoids (canary-xanthophylls A and B and lutein [50,54]). The coloration of the tail feathers is affected by coccidiosis during moult [51].

**Rump colour:** No data.

**Head colour:** No data.

**Back colour:** No data.

# References

# 1. Chantrey DF, Workman L (1984) Song and Plumage Effects on Aggressive Display by the European Robin *Erithacus rubecula*. Ibis 126: 366-371.

# 2. Faivre B, Gregoire A, Preault M, Cezilly F, Sorci G (2003) Immune activation rapidly mirrored in a secondary sexual trait. Science 300: 103-103.

# 3. Faivre B, Preault M, Thery M, Secondi J, Patris B, et al. (2001) Breeding strategy and morphological characters in an urban population of blackbirds, Turdus merula. Animal Behaviour 61: 969-974.

# 4. Preault M, Deregnaucourt S, Sorci G, Faivre B (2002) Does beak coloration of male blackbirds play a role in intra and/or intersexual selection? Behavioural Processes 58: 91-96.

# 5. Bright A, Waas JR (2002) Effects of bill pigmentation and UV reflectance during territory establishment in blackbirds. Animal Behaviour 64: 207-213.

# 6. Faivre B, Preault M, Salvadori F, Thery M, Gaillard M, et al. (2003) Bill colour and immunocompetence in the European blackbird. Animal Behaviour 65: 1125-1131.

# 7. Bright A, Waas JR, King CM, Cuming PD (2004) Bill colour and correlates of male quality in blackbirds: an analysis using canonical ordination. Behavioural Processes 65: 123-132.

# 8. Hegyi G, Szigeti B, Török J, Eens M (2007) Melanin, carotenoid and structural plumage ornaments: information content and role in great tits *Parus major*. Journal of Avian Biology 38: 698-708.

# 9. Ferns PN, Hinsley SA (2004) Immaculate tits: head plumage pattern as an indicator of quality in birds. Animal Behaviour 67: 261-272.

# 10. Woodcock EA, Rathburn MK, Ratcliffe LM (2005) Achromatic plumage reflectance, social dominance and female mate preference in black-capped chickadees (Poecile atricapillus). Ethology 111: 891-900.

# 11. Doucet SM, Mennill DJ, Montgomerie R, Boag PT, Ratcliffe LM (2005) Achromatic plumage reflectance predicts reproductive success in male black-capped chickadees. Behavioral Ecology 16: 218-222.

# 12. Lucas AM, Stettenheim PR (1972) Avian anatomy - Integument. Washington D. C.: US Department of Agriculture.

# 13. Partali V, Liaaen-Jensen S, Slagsvold T, Lifjeld JT (1987) Carotenoids in food chain studies-II. The food chain of *Parus spp.* monitored by carotenoid analysis. Comp Biochem Physiol 87B: 885-888.

# 14. Slagsvold T, Lifjeld JT (1985) Variation in plumage colour of the Great tit *Parus major* in relation to habitat, season and food. J Zool Lond (A) 206: 321-328.

# 15. Hõrak P, Ots I, Vellau H, Spottiswoode C, Møller AP (2001) Carotenoid-based plumage coloration reflects hemoparasite infection and local survival in breeding great tits. Oecologia 126: 166-173.

# 16. Horak P, Surai PF, Ots I, Møller AP (2004) Fat soluble antioxidants in brood-rearing great tits *Parus major*: relations to health and appearance. Journal of Avian Biology 35: 63-70.

# 17. Mand R, Tilgar V, Moller AP (2005) Negative relationship between plumage colour and breeding output in female great tits, *Parus major*. Evolutionary Ecology Research 7: 1013-1023.

# 18. Senar JC, Figuerola J, Domènech J (2003) Plumage coloration and nutritional condition in the great tit *Parus majo*r: the roles of carotenoids and melanins differ. Naturwissenschaften 90: 234-237.

# 19. Fitze PS, Richner H (2002) Differential effects of a parasite on ornamental structures based on melanins and carotenoids. Behavioral Ecology 13: 401-407.

# 20. Galvan I, Sanz JJ (2006) Feather mite abundance increases with uropygial gland size and plumage yellowness in Great Tits *Parus major*. Ibis 148: 687-697.

# 21. Dufva R, Allander K (1995) Intraspecific variation in plumage coloration reflects immune response in great tit (*Parus major*) males. Functional Ecology 9: 785-789.

# 22. Isaksson C, Uller T, Andersson S (2006) Parental effects on carotenoid-based plumage coloration in nestling great tits, *Parus major*. Behavioral Ecology and Sociobiology 60: 556-562.

# 23. Quesada J, Senar JC (2007) The role of melanin- and carotenoid-based plumage coloration in nest defence in the Great Tit. Ethology 113: 640-647.

# 24. Delhey K, Kempenaers B (2006) Age differences in blue tit *Parus caeruleus* plumage colour: within-individual changes or colour-biased survival? Journal of Avian Biology 37: 339-348.

# 25. Sheldon BC, Andersson S, Griffith SC, Örnborg J, Sendecka J (1999) Ultraviolet colour variation influences blue tit sex ratios. Nature 402: 874-877.

# 26. Griffith SC, Örnborg J, Russell AF, Andersson S, Sheldon BC (2003) Correlations between ultraviolet coloration, overwinter survival and offspring sex ratio in the blue tit. J Evol Biol 16: 1045-1054.

# 27. Korsten P, Lessells CM, Mateman AC, van der Velde M, Komdeur J (2006) Primary sex ratio adjustment to experimentally reduced male UV attractiveness in blue tits. Behavioral Ecology 17: 539-546.

# 28. Delhey K, Peters A, Johnsen A, Kempenaers B (2007) Brood sex ratio and male UV ornamentation in blue tits (*Cyanistes caeruleus*): correlational evidence and an experimental test. Behavioral Ecology & Sociobiology In press.

# 29. Dreiss A, Richard M, Moyen F, White J, Moller AP, et al. (2006) Sex ratio and male sexual characters in a population of blue tits, Parus caeruleus. Behavioral Ecology 17: 13-19.

# 30. Limbourg T, Mateman AC, Andersson S, Lessells CM (2004) Female blue tits adjust parental effort to manipulated male UV attractiveness. Proceedings of the Royal Society of London Series B-Biological Sciences 271: 1903-1908.

# 31. Johnsen A, Delhey K, Schlicht E, Peters A, Kempenaers B (2005) Male sexual attractiveness and parental effort in blue tits: an experimental test of the differential allocation hypothesis. Animal Behaviour 70: 877-888.

# 32. Szigeti B, Torok J, Hegyi G, Rosivall B, Hargitai R, et al. (2007) Egg quality and parental ornamentation in the blue tit Parus caeruleus. Journal of Avian Biology 38: 105-112.

# 33. Hunt S, Bennett ATD, Cuthill IC, Griffiths R (1998) Blue tits are ultraviolet tits. Proc R Soc Lond B 265: 451-455.

# 34. Hunt S, Cuthill IC, Bennett ATD, Griffiths R (1999) Preferences for ultraviolet partners in the blue tit. Animal Behaviour 58: 809-815.

# 35. Andersson S, Örnborg J, Andersson M (1998) Ultraviolet sexual dimorphism and assortative mating in blue tits. Proc R Soc Lond B 265: 445-450.

# 36. Delhey K, Johnsen A, Peters A, Andersson S, Kempenaers B (2003) Paternity analysis reveals opposing selection pressures on crown coloration in the blue tit (*Parus caeruleus*). Proc R Soc Lond B 270: 2057-2064.

# 37. Delhey K, Peters A, Johnsen A, Kempenaers B (2007) Fertilization success and UV ornamentation in blue tits *Cyanistes caeruleus*: correlational and experimental evidence. Behavioral Ecology In press.

# 38. Peters A, Delhey K, Goymann W, Kempenaers B (2006) Age-dependent association between testosterone and crown UV coloration in male blue tits (*Parus caeruleus*). Behavioral Ecology and Sociobiology 59: 666-673.

# 39. Foerster K, Delhey K, Johnsen A, Lifjeld JT, Kempenaers B (2003) Females increase offspring heterozygosity and fitness through extra-pair matings. Nature 425: 714-717.

# 40. Harper DGC (1999) Feather mites, pectoral muscle condition, wing length and plumage coloration of passerines. Animal Behaviour 58: 553-562.

# 41. Alonso-Alvarez C, Doutrelant C, Sorci G (2004) Ultraviolet reflectance affects male-male interactions in the blue tit (*Parus caeruleus ultramarinus*). Behavioral Ecology 15: 805-809.

# 42. Korsten P, Vedder O, Szentirmai I, Komdeur J (2007) Absence of status signalling by structurally based ultraviolet plumage in wintering blue tits (Cyanistes caeruleus). Behavioral Ecology and Sociobiology 61: 1933-1943.

# 43. Poesel A, Dabelsteen T, Darden SK, Delhey K, Peters A (2007) Territorial responses of male blue tits, Cyanistes caeruleus, to UV-manipulated neighbours. Journal of Ornithology 148: 179-187.

# 44. Hidalgo-Garcia S (2006) The carotenoid-based plumage coloration of adult Blue Tits *Cyanistes caeruleus* correlates with the health status of their brood. Ibis 148: 727-734.

# 45. Senar JC, Figuerola J, Pascual J (2002) Brighter yellow blue tits make better parents. Proc R Soc Lond Ser B-Biol Sci 269: 257-261.

# 46. Svensson E, Merilä J (1996) Molt and migratory condition in blue tits: a serological study. Condor 98: 825-831.

# 47. Merilä J, Sheldon BC (1999) Testis size variation in the greenfinch *Carduelis chloris*: relevance for some recent models of sexual selection. Behavioral Ecology & Sociobiology 45: 115-123.

# 48. Merilä J, Sheldon BC, Lindstrom K (1999) Plumage brightness in relation to haematozoan infections in the greenfinch Carduelis chloris: Bright males are a good bet. Ecoscience 6: 12-18.

# 49. Lindström K, Lundström J (2000) Male greenfinches (*Carduelis chloris*) with brighter ornaments have higher virus infection clearance rate. Behavioral Ecology and Sociobiology 48: 44-51.

# 50. Stradi R, Celentano G, Rossi E, Rovati G, Pastore M (1995) Carotenoids in Bird Plumage.1. The Carotenoid Pattern in a Series of Palearctic Carduelinae. Comparative Biochemistry and Physiology B-Biochemistry & Molecular Biology 110: 131-143.

# 51. Horak P, Saks L, Karu U, Ots I, Surai PF, et al. (2004) How coccidian parasites affect health and appearance of greenfinches. Journal of Animal Ecology 73: 935-947.

# 52. Saks L, Ots I, Horak P (2003) Carotenoid-based plumage coloration of male greenfinches reflects health and immunocompetence. Oecologia 134: 301-307.

# 53. Aguilera E, Amat JA (2007) Carotenoids, immune response and the expression of sexual ornaments in male greenfinches (*Carduelis chloris*). Naturwissenschaften 94: 895-902.

# 54. Saks L, McGraw KJ, Horak P (2003) How feather colour reflects its carotenoid content. Funct Ecol 17: 555-561.
